# Designing and validating the learner autonomy perception questionnaire

**DOI:** 10.1016/j.heliyon.2021.e06831

**Published:** 2021-04-21

**Authors:** Son Van Nguyen, Anita Habók

**Affiliations:** a Doctoral School of Education, University of Szeged, Hungary; b Institute of Education, University of Szeged, Hungary

**Keywords:** Learner autonomy, Questionnaire, Validation, Perception, English language learning (ELT)

## Abstract

The present study aimed to design and validate a questionnaire to investigate students' perceptions of learner autonomy in the context of Vietnamese tertiary education. The questionnaire was adapted from various well-established scales in the literature, and then the back-translation method was used to produce a version in Vietnamese. After the questionnaire development process, 1,565 non-English majors at seven different tertiary institutions in Vietnam voluntarily participated in the study and completed the questionnaire. Evidence of reliability and validity was provided for the instrument using SPSS Version 24, SmartPLS 3, and SPSS AMOS. Reliability was evaluated using Cronbach's alpha, composite reliability (CR), rho_A value, and average inter-item correlations. Validity was substantiated using Messick's framework of validity (1995). This entailed five different aspects: content, substantive, structural, external, and consequential. The results indicated that reliability reached adequate values and the aspects of validity were mostly confirmed. The questionnaire, therefore, was suited to exploring how students perceive learner autonomy, but it requires more validation for future use in the other contexts.

## 1 Introduction

Learner autonomy (LA) is marked by “a readiness to take charge of one's own learning in the service of one's needs and purposes. This entails a capacity and willingness to act independently and in cooperation with others as a socially responsible person” (Dam, 1995, p. 1), widely known as Bergen definition. LA has represented an answer to the challenges facing the 21^st^-century education to potentially satisfy the demands of the labor market (Blidi, 2017). LA itself is a principal learning outcome of higher education across many nations in the world (Henri et al., 2017) and developing LA is a pedagogical approach to promoting lifelong learning (Dam, 2012). Specifically, LA is “an educational goal of teaching English as a foreign language” (Teng, 2019, p. 1), with its role having been declared as one of the “essentials for successful language learning” in Farrell and Jacobs' book (2010) of the same title. Farrell and Jacobs (2010) properly clarified the reasons why LA has become the most important of eight essentials for successful language learning. Indeed, LA has been discussed in English language teaching and learning for approximately forty years with an increasing number of publications on LA (Benson, 2009; Little, 2020; Ou, 2017; Reinders, 2011). Nevertheless, there is likelihood that in Vietnam, it is not a familiar concept, and thus, there is only a small number of studies on LA. The research done before is on language instructors' and English majors' perceptions of LA and their practices (e.g. Bui, 2016; Dang, 2012; Le, 2013; N. T. Nguyen, 2014) and on strategies to nurture LA (Cao, 2018; Hoang and Nguyen, 2010; Humphreys and Wyatt, 2014; L. T. C. Nguyen, 2009; N. T. Nguyen, 2012; Phan, 2015; Tran, 2005). Those studies indicated that there was a relationship between LA and language proficiency. Also, there was a difference between learners' perceptions and their autonomous performances. They mainly demonstrated reactive autonomy proposed by Littlewood (1999). However, perceptions of LA among non-English majors have not been properly studied. Additionally, there is a lack of survey instruments to explore perceptions of LA in many aspects. Moreover, it is nearly improbable that the questionnaires explicitly report on their formulation, development, and validation. Therefore, this study has been conducted to address these gaps. The primary purpose of this study has been as follows: (a) to construct a data collection instrument named as the learner autonomy perception questionnaire (LAPQ), which uses data collected from a sample of 1,565 English learners from several Vietnamese higher education institutions and (b) to perform validity and reliability analyses of the referenced questionnaire.

## 2 Theoretical framework

To conceptualize LA, the theoretical underpinning of this study has its roots in the Bergen definition aforementioned. There are three main reasons to choose this definition.

Firstly, it is in line with Holec (1981) and Benson (2011) seminal definitions. They described LA as a construct of capacity, which refers to the psychological perspective of LA. This capacity is typified by an ability to make decisions about learning, requiring three principles: (a) a certain amount of metacognitive knowledge about the learners themselves, the context, the subject, and the learning prfocess; (b) conscious awareness of this knowledge; and (c) conscious reflection on learning. It also requires the usage of metacognitive strategies such as planning, goal setting, monitoring, self-assessment, evaluation, and using learning resources (Sinclair, 2009). Secondly, it values the significance of willingness or readiness as regards LA Indeed LA “is a construct of capacity which is operationalized when willingness is present” (Sinclair, 2009, p. 185). That readiness facilitates successful implementation of LA-based programs by guiding curriculum development and classroom practice (Chan et al., 2002; Lin and Reinders, 2019). Also, investigating willingness for LA enables researchers to leave from the culturist view of LA (Yıldırım, 2012). Thirdly, this definition is suited to the context of Vietnam. Specifically, it does not mention the power in the political perspective of LA which highlights learners’ power and freedom to make informed choices in their learning process (Bui, 2018; Oxford, 2003; Pennycook, 2013). It is a fact that both teachers and students in Vietnam cannot choose or take control of their learning content which is one of three dimensions of control over learning (Huang and Benson, 2013) because they must follow and implement the learning content predetermined by institutional and national curricula.

The present study stands on the pole of the quintessential strategy which focuses on analyzing components of a construct (see more in Benson, 2009). Hence, the study will conceptualize its view of LA on the basis of its two most essential components: willingness and capacity (Hsu, 2005; Littlewood, 1996, 1999; Sinclair, 2000a, b). Students need willingness as well as capacity so that they can take on responsibility for their language learning, and enhance LA. The explanation about these components will be as follows.

### 2.1 Willingness

As the time went by, the researchers thickened and deepened the literature on LA conceptualization by taking more components into account. To be more specific, during the period of advent in the 1980s, responsibility and decision-making became the most popular (Hsu, 2005). In the next decade of the 1990s, several individual attributes were added such as attitudes, willingness, and confidence, which are named “affective factors” by Tran (2005). Willingness is emphasized to be one of the most significant components of LA (Hsu, 2005). Affective factors like willingness play an important role in the development of LA (Cotterall, 1995 Hsu, 2005; Le, 2013; Lin and Reinders, 2019; Ming and Alias, 2007; Nguyen, 2011; Sinclair, 2000a; Wenden, 1991). As regardless of their capacity, students will not enhance their LA if they are not willing to take charge of their learning (Sinclair, 2000b). Willingness to engage in autonomous learning consists of two components, namely beliefs about teacher's role and motivation (Chan et al., 2002; Dixon, 2011; Hsu, 2005) which will be explained below.

#### 2.1.1 Beliefs about teacher's role

Learners are expected to be aware of the roles of teachers and their own roles because their beliefs as regards their role may strongly influence their exercise of responsibility in or out of class and their readiness to learn English autonomously. This point is strongly supported by Chan et al. (2002), Cotterall (1995, 1999), Dişlen (2011), Hozayen (2011), Le (2013), Mousavi Arfae (2017), Swatevacharkul (2009), and Tomita and Sano (2016). It can be argued that students' beliefs about language learning are underpinned by the behaviors of teachers. Those who believe that teachers are facilitators of learning are ready for autonomous learning; by contrast, learners who think that teachers should tell them what to do, offer help, and explain everything are not yet ready for LA (Rungwaraphong, 2012). Hence, students' expectations of teacher authority can impede teachers from transferring responsibility to them (Cotterall, 1995). There is a strong conviction that learners' beliefs about their own roles and their teacher's roles will make a great contribution to their willingness to embrace LA.

#### 2.1.2 Motivation

There exists an argument among the researchers toward the question “Which one comes first and which one depends on which one, motivation or autonomy?“. Lamb (2011) convincingly elucidates the question above (Ushioda, 2011, 2014). Specifically, he starts with a metaphor of “which came first, the worm or the cocoon?“, and then distinguishes two senses of autonomy, one of which refers to taking responsibility for manipulating one's own learning. This notion of autonomy is learner autonomy or language learner autonomy in the words of Little (2007) (Ushioda, 2011, 2014). It entails a willingness for responsibility and a capacity for “detachment, critical reflection, decision-making, and independent action” (Little, 1991, p. 4). Accordingly, an autonomous language learner can employ metacognitive skills and strategic thinking processes to overcome challenges in language learning. However, to exercise such metacognitive skills and ability, willingness or motivation is a prerequisite, so this sense of autonomy depends on motivation. That is, if learners have motivation or willingness, they will exercise their autonomy to learn the language beyond the basic requirements (Lamb, 2011; Ushioda, 2011). As a result, learners are expected to be motivated to develop LA.

Autonomous learners are motivated and reflective learners, which results in efficient and effective learning (Little, 1991). Further, the development of motivation is the locomotive of LA, conducive to the development of LA (Hsu, 2005). Indeed, motivation is essential in promoting autonomous learning (Benson, 2007; Hu and Zhang, 2017; Liu, 2015). It has been seen as one of the components of LA (Chan et al., 2002; Dixon, 2011; Henri et al., 2017; Hsu, 2005; Le, 2013; Littlewood, 1996; Macaskill and Taylor, 2010; Nguyen, 2008, 2009; Swatevacharkul, 2009; Tassinari, 2012; Ushioda, 1996; Zarei and Elakaei, 2012). We believe that motivation is one of the tools with which learners equip themselves to enter the learning situation and enhance LA. We agree with Littlewood (1996), Hsu (2005), and Swatevacharkul (2009) that motivation is best conceptualized when it is subsumed under the notion of willingness.

### 2.2 Capacity

**Figure 1:**
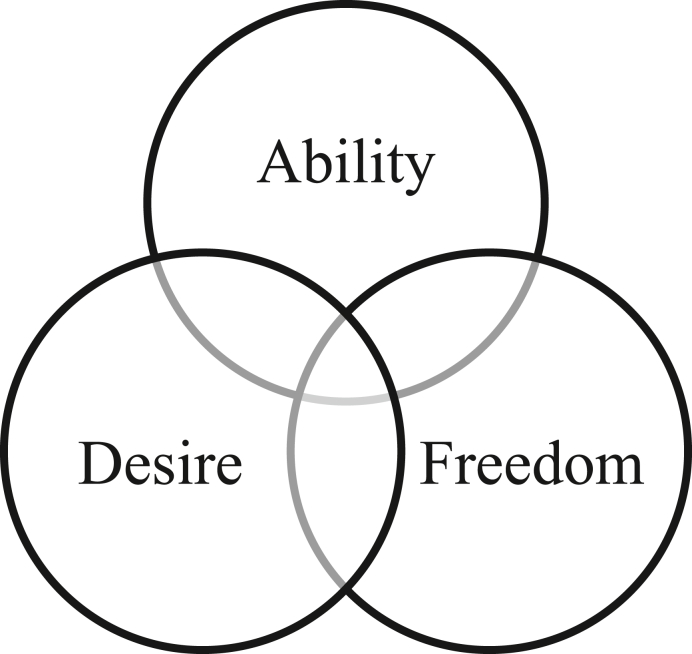
Venn diagram on *What is capacity of LA?* (Adapted from Huang and Benson, 2013).

As for the concept of capacity, we will adopt the conceptual framework developed by Huang and Benson (2013). Accordingly, capacity consists of ability, desire, and freedom (see Figure 1). These are discussed in the following sections.

#### 2.2.1 Ability

Ability represents knowledge and skills related to studying and language (Benson, 2013). This study is not aimed at measuring linguistic knowledge or skills, or knowledge of English, nor is it designed to examine English language skills, such as reading or writing, so knowledge of English components (i.e., pronunciation, grammar, and vocabulary) and English skills are not taken into consideration.

Knowledge of studying is nothing but metacognitive knowledge. Generally, like declarative knowledge, it differentiates between knowledge about person, task, and strategy (Flavell, 1979; Veenman, 2011). Knowledge of task includes characteristics of task and when to use a strategy, whereas knowledge of strategy refers to how to use a strategy and why. In language learning, metacognitive knowledge is specified to comprise knowledge of self as a language learner; knowledge of the socio-cultural, political, and physical learning context; knowledge of the subject matters; and knowledge of language learning processes (Sinclair, 2000a). The three aspects of metacognitive knowledge are “(a) awareness of their strengths and weaknesses in relation to the tasks; (b) an understanding of the tasks they are engaged in; and (c) knowledge of strategies which can help them undertake such tasks” (Cotterall, 2009, p. 88). Drawing on insights from these views, this study argues that the classifications developed by Sinclair (2000a) and Cotterall (2009) are consistent with the influential definition of metacognitive knowledge offered by Flavell (1979). This consistency is illustrated in Table 1.

**Table 1:** Metacognitive knowledge in language learning.

Flavell (1979)	Sinclair (2000a)	Cotterall (2009)
Knowledge of person	Knowledge of self as a language learner	Awareness of their strengths and weaknesses in relation to tasks
Knowledge of task	Knowledge of socio-cultural, political, and physical learning context; Knowledge of subject matter	Understanding of tasks they are engaged in
Knowledge of strategy	Knowledge of language learning processes	Knowledge of strategies which can help them undertake such tasks

Study skills refer to planning, monitoring, and evaluating (Huang and Benson, 2013), which can be technically summarized as metacognitive skills (Nguyen, 2009). It is consitent with Little (1991, 2020), and Murray (2014) arguments that those skills are included in the capacity of autonomy. They are closely related to the improvement of LA (García Magaldi, 2010; Nguyen and Gu, 2013; Wenden, 1991) and even central to autonomous ability (Hsu, 2005). Without them, students basically do not have directions and ability to monitor their progress, attainment, and future paths (O'Malley et al., 1985). Hence, with regard to LA in language learning, ability is characterized by metacognitive knowledge and metacognitive skills.

#### 2.2.2 Desire

Informed by specific purposes, desire is how intensely learners intend to learn English, and complete a learning task (Benson, 2013; Huang and Benson, 2013). Those purposes, as we argue, should be culturally suitable in the context of Vietnam where English language learning happens, and students' desire should be expressed by specific thoughts and actions. To exemplify, if English courses were not conducted at university, students would attend English lessons somewhere else. The university represents the context in this case. The specific purpose may be interest in English language, university's requirements, or future job prospects. Those students show their desire by obtaining lessons in other places. Generally speaking, that action can be seen as the embodiment of LA.

#### 2.2.3 Freedom

Freedom is denoted as “the degree to which learners are “permitted” to control their learning, either by specific agents in the learning process, or more generally by the learning situations in which they find themselves” (Huang and Benson, 2013, p. 9). The researchers (e.g., Lamb, 2009) believe that freedom can be demonstrated through a variety of observable activities which learners are allowed to do and which they do in reality to take charge of their own learning. For example, students have chances to ask their English teachers when they do not understand something or make suggestions to English teachers. The real autonomous activities they do can be writing emails or Facebook statuses, or listening to English frequently (Chan et al., 2002). However, it is noteworthy that in Vietnam, due to the prescribed syllabus and program, it is impossible for students to choose learning materials, and learning activities. They are not allowed to decide on what they would like to learn. In other words, control over learning content is not accessible to EFL learners.

**Figure 2:**
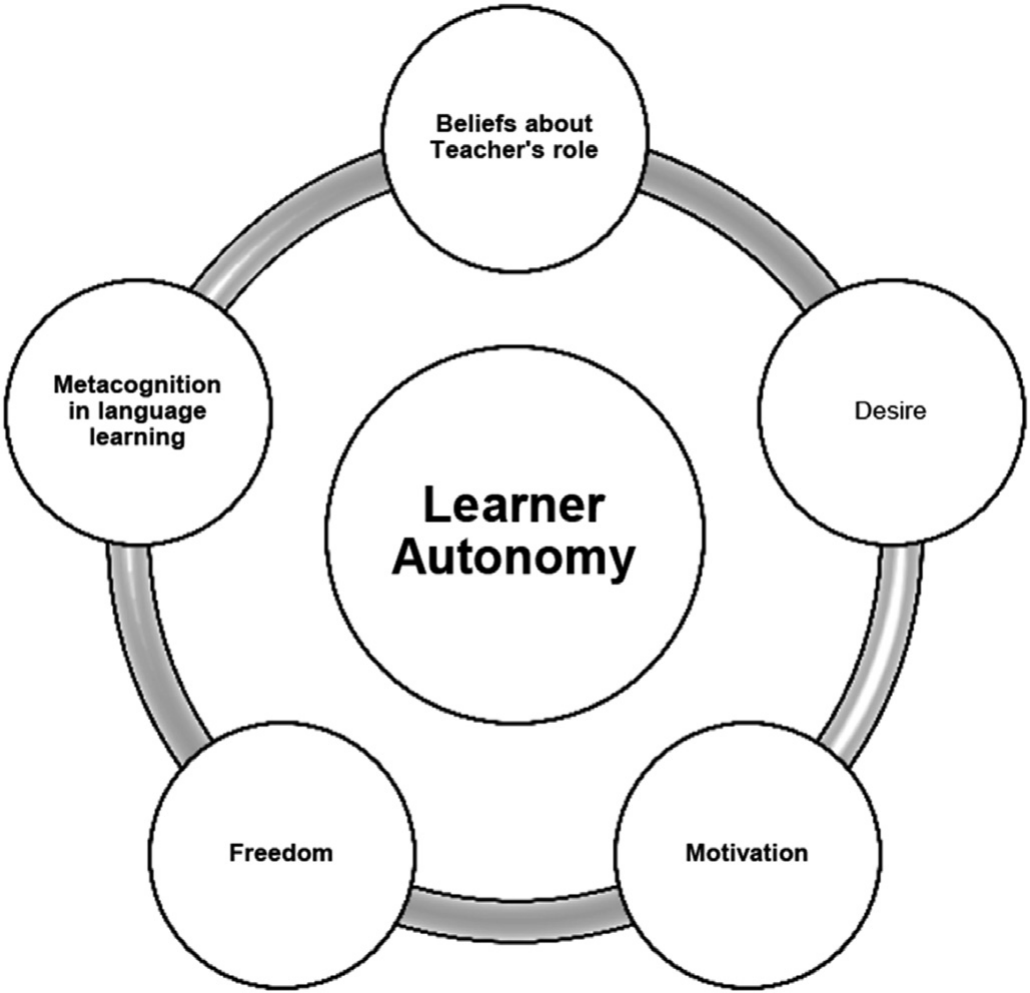
Conceptualization of Learner Autonomy (Nguyen and Habók, 2020, p. 126).

In conclusion, willingness and capacity are important components for students to take over responsibility for learning English. Willingness includes two factors, namely beliefs about teacher's role and motivation. Capacity comprises ability, which encompasses metacognitive knowledge as well as metacognitive skills, desire, and freedom. To conceive LA, metacognitive knowledge and metacognitive skills are termed into metacognition as a component of LA. This is consistent with Dixon (2011), Haque (2015), and Reinders (2011) which indicate that metacognition is a crucial part of LA. As a result, LA in our study is characterized by four components: beliefs about teacher's role, motivation, desire, metacognition, and freedom. This understanding is illustrated in Figure 2 (see more in Nguyen and Habók, 2020). In this study, we conceptualize LA as students' willingness and capacity to take control of their foreign language learning. The former manifests itself in learners' beliefs about teacher's role and motivation to learn languages. Importantly, we believe learners must possess metacognitive knowledge and metacognitive skills to effectively acquire English language and fulfill their language needs in the world of the fourth industrial revolution which is changing rapidly. To that end, we argue they must also possess desire as well as a certain freedom to involve themselves in the language teaching and learning process. The classsification of components of LA in this study is basically aligned with that in previous studies such as Cooker (2012), Dixon (2011), and Tassinari (2012). The aspects as well as dimensions of LA are action-oriented (e.g., freedom and metacognitive skills), cognitive (e.g., beliefs), metacognitive (e.g., metacognitive knowledge), affective and motivational (e.g., motivation and desire). They theoretically positively interact to each other and show a balance themselves in different contexts, which typifies a characteristic of LA in a dynamic model (see more in Tassinari, 2012; Tassinari, 2015; Tassinari, 2018). This provides theoretical fundamentals for discriminant and convergent validity later discussed in this study.

## 3 Methodological framework

We employed Messick (1995) framework of validity for instrument validation in this study. Validity is defined as the property of the meaning of the test scores and regarded as a unified construct framework which integrates dimensions of content, criteria, and consequences (Messick, 1995). Messick (1995) adds that as a unitary construct, validity can be differentiated into distinguishable aspects to “provide a means of addressing functional aspects of validity that help disentangle some of the complexities inherent in appraising the appropriateness, meaningfulness, and usefulness of score inferences” (p. 5). Six aspects highlighted include content, substantive, structural, generalizability, external, and consequential. These aspects serve as a framework of general validity criteria and standards for educational and psychological measures (Messick, 1989), which will be described as follows. This study appraised five out of six aspects of validity and excluded generalizability because we did not gain access to other populations to generalize the interpretation across settings and groups.

The content aspect consists of evidence of content relevance, representativeness, and technical quality, which is usually evaluated by experts (Wang and Bai, 2017). The substantive aspect focuses on theoretical rationales for consistencies of responses to items. The generalizability aspect highlights how properties and interpretations generalize across tasks, contexts, and groups. The consequential aspect appraises the implications of score interpretations and test use.

The structural aspect examines how the internal structure is reflected in the scores. Exploratory factor analysis (EFA) and confirmatory factor analysis (CFA) were employed to evaluate this aspect of validity. EFA enables researchers to examine the relationships between latent variables and observed ones. There are several factors in EFA that call for research attention. They are the Kaiser–Meyer–Olkin measure of sampling adequacy and Bartlett's test of sphericity, extraction, rotation, variance, and particularly, results in parallel analysis (PA). PA developed initially by Horn (1965) and further by Glorfeld (1995) is considered one of the precise methods to determine the number of factors to retain; however, it has been under-investigated by researchers (Çokluk & Koç;ak, 2016; Hayton et al., 2004; Henson and Roberts, 2006; Worthington and Whittaker, 2006). In this method, eigenvalues extracted from a random dataset are compared to those extracted from the original dataset and these two datasets are parallel (Loewen and Gonulal, 2015; O'Connor, 2000). The factor is retained if the eigenvalue generated from the actual data is higher than the corresponding eigenvalue from the parallel data (Dinno, 2009; Franklin et al., 1995; O'Connor, 2000). CFA uses a certain set of goodness-of-fit indices, such as Chi-square χ^2^, the comparative fit index (CFI), the Tucker–Lewis index (TLI), the root mean square error of approximation (RMSEA), the normed fit index (NFI), the root mean square residual covariance (RMS_theta), and the standardized root mean square residual (SRMR). It is advisable for researchers to calculate and report different fit indices in their studies because of the lack of global agreement on the acceptable values of those indices (Martens, 2005; Teo et al., 2013). In this research, model fit was assessed on the basis of four absolute fit indices: Chi-square, SRMR, RMSEA, and RMS_theta, along with three incremental indices including TLI, NFI, and CFI in comparative fit. Specifically, as regards absolute fitting, a significant value of χ^2^ highlights the inappropriateness of the proposed model to the sample data (Teo et al., 2013). However, this very much depends on the sample size (Cangur and Ercan, 2015), so Chi-square cannot be regarded as the only indicator of model fit. To remedy this sample dependence, Glynn et al. (2011) divide Chi-square by the degree of freedom (χ^2^/d.f.); as a result, the researchers arrive at a normed Chi-square value (Kline, 2015). It should be more than 2.0 (Tabachnick and Fidell, 2007) and less than 5.0 (Wheaton et al., 1977). SRMR refers to the degree of error due to estimation of the specified model, and its adequate level of model fit was no more than 0.06 (Teo, 2013) or 0.08 (Hu and Bentler, 1999). RMSEA “corrects the tendency of the χ^2^ to reject models with large same size or number of variables”, and should be 0.05 or less with a confidence level of 95% (Teo et al., 2013, p. 15). Both SRMR and RMSEA are not greatly affected by sample size (Chen, 2007; Marsh et al., 2004). RMS_theta signifies the extent of correlations between the outer model residuals (Lohmöller, 1989), and Henseler et al. (2014) suggest that a well-fitting model has a RMS_theta value below 0.12. Turning to comparative fitting, NFI, proposed by Bentler and Bonett (1980), calculates Chi-square value and compares it to a meaningful standard value.^1^ NFI falls between 0 and 1, and the more it approaches 1, the better the model fit. The preferable level is 0.9. TLI, also known as Bentler–Bonett non-normed fit index (NNFI), is used to compare our model to the baseline model, and a well-fitting model has a TLI closer to 1.0. The higher the TLI value, the better the model. CFI assesses the lack of fit of the proposed model over a null model (Kline, 2015). Teo et al. (2013) comment that CFI is popular due to its strength, thus indicating that CFI is not sensitive to the complexity of the model. The cut-off value of CFI is 0.9 to achieve goodness of fit (Basu and Miroshnik, 2019; Hooper et al., 2008); however, the cut-off is not perfect all the time despite being widely used (Teo, 2013). In this study, EFA is of paramount importance because it enables us to consider which items to exclude from the questionnaire for better reliability and validity. It also provides a comprehensive overview of the questionnaire structure. CFA allows us to inspect the hypothesized model of LA. Hence, if it were not confirmed, more validation analyses would be necessary.

The external aspect refers to convergent and discriminant evidence. The former demonstrates the degree to which items are related to each other, and it is confirmed by average variance extracted (AVE), factor loadings, and CR which is calculated by the total amount of true scale variance divided by the total variance scale score (Brunner and Süß, 2005). CR uses standardized loadings to explore the reliability of scales (Chin, 2010; Raykov, 1997). Specifically, AVE should be higher than the threshold of 0.5. However, if AVE is lower than that threshold, and CR is higher than 0.6, convergent validity will be acceptably established (Fornell and Larcker, 1981). AVE is criticized for often being too strict, and convergent validity can be evaluated by CR only (Malhotra and Dash, 2011). The latter provides evidence of whether items on a scale can be differentiated from those on other scales. It is assessed on the basis of Fornell and Larcker (1981) criterion, cross-loadings, or heterotrait-monotrait ratio of correlations (HTMT). According to Fornell and Larcker's seminal article (1981), discriminant validity can be confirmed if the square root of AVE is higher than the correlation of one latent variable with other latent variables. In terms of cross-loadings, correlation with another latent variable should not be better than that with its own latent variable (Garson, 2016). HTMT is denoted as “the average of the heterotrait-heteromethod correlations (i.e., the correlations of indicators across constructs measuring different phenomena), relative to the average of the monotrait-heteromethod correlations (i.e., the correlations of indicators within the same construct)” (Henseler et al., 2015, p. 121). Henseler et al. (2015) offer two ways of using HTMT for discriminant validity. The first is employing HTMT as a criterion compared to a threshold. The second involves HTMT serving as a statistical test. We applied the first in this study, and the threshold was set at 0.9 (Gold et al., 2001; Teo et al., 2008). The HTMT ratio should thus be under 0.9 so that discriminant validity can be accepted.

After we obtained the revised version of the questionnaire, internal consistencies in the instrument and each scale were examined with Cronbach's alpha. Also, we employed CR to analyze reliability. Additionally, reliability was assessed by rho_A, which evaluates the weight of the constructs, not their loadings, and is remarked by “the off-diagonal elements of a latent variable's indicator correlation matrix are reproduced as well as possible in a least squares sense” (Dijkstra and Henseler, 2015a; Dijkstra and Henseler, 2015b, p. 300). Moreover, average inter-item correlations among the sub-scales were used to investigate whether the items are related to the other items in the scale and whether they assess the same construct (Cohen and Swerdlik, 2005). The suggested value of the correlation was between 0.2 and 0.4 (Piedmont, 2014) or even between 0.166 and 0.830 (Ferrell et al., 2000).

## 4 Methods

### 4.1 Participants

A sample of 1,565 university students in total were voluntarily recruited from seven universities in Hanoi, Vietnam. Those students were learning English as a minor part in their curriculum and studying the following subjects: information technology (IT) (n = 339; 21.7%), economics (n = 184; 11.8%), civil engineering (n = 124; 7.9%), electrical and electronic engineering (n = 259; 16.5%), mechanical engineering (n = 191; 12.2%), law (n = 188; 12%), and various other fields (n = 280; 17.9%).

The sample consisted of 62% students in their second year (n = 971), 23.7% third-year students (n = 371), 11.9% students in their fourth year (n = 186), and 2.4% fifth-year students (n = 37). The respondents reported an average of over 11 years of formal instruction in English (11.7, SD = 1.4). Among the students, 62.2% were male (n = 974), and 37.8% were female (n = 591). As regards students' place of residence within Vietnam, Hanoi is the most popular city, with 28.7% of the students coming from there (n = 449), followed by Nam Dinh Province (n = 172; 11%) and Thai Binh Province (n = 109; 7%). The participants hailed from 34 out of 64 provinces in Vietnam, thus showing a geographic diversity. They have studied English at universities for at least one semester in order to make sure that they were more familiar with and experienced in the tertiary language education environment than their peers in their first year. This enabled them to reflect on themselves and provide in-depth information on their learner autonomy. The participants’ diverse demographic background information might enable the researchers to improve the generalizability of the results to a broader population (Tran et al., 2013). The description of the participants can be found in a report on research project by Nguyen and Habók (2020).

As indicated by the literature (e.g. Cabrera-Nguyen, 2010; Fokkema and Greiff, 2017; Hinkin, 1998; Izquierdo et al., 2014; Worthington and Whittaker, 2006), EFA and CFA should be carried out on different samples. Therefore, the sample above was split into two sub-samples in a random way. The first sub-sample included responses of 780 students to conduct EFA and the other one of 785 students was used to perform CFA.

### 4.2 Instrument

To develop the questionnaire, we consulted the relevant literature and critically investigated the accredited questionnaires. We borrowed and modified items from frequently used questionnaires that have been established with psychometrically sound properties. This is an important step to generate items, thus aiding in the improvement of the validity and reliability evidence (Dörnyei, 2010). After the literature was systematically reviewed, an initial pool of 87 self-reported items was compiled (see Appendix A). The details of numbers of items, and their sources are presented in Table 2. Eight items were shared by Chan et al. (2002) and Le (2013); seven items were used by both Hsu (2005) and Swatevacharkul (2009); and five items were employed by Cotterall (1999) and Hsu (2005).

**Table 2:** Sources of items in the pool.

Number of items	Sources
37	Hsu (2005)
19	Dang (2012), Yang (2007)
18	Chan et al. (2002)
08	Le (2013)
07	Cotterall (1999)
07	Swatevacharkul (2009)
03	Ming and Alias (2007)
02	Dixon (2011)
02	Cotterall (1995)
04	Researchers in this study

The items and their sources can be found in Appendix B. The questionnaire employed a five-point Likert scale ranging from 1 (*strongly disagree*) to 5 (*strongly agree*). The items were arranged in random order, instead of a scale-by-scale order, so that the data collected could be more objective and not scale-oriented.

The *beliefs about teacher's role* (BTR) scale contains eleven items adapted from Ming and Alias (2007), Chan et al. (2002), and Le (2013). There are eight items on the *motivation* (M) scale. We added item 65 to refer to one dimension of motivation to learn English. The others were adapted from Hsu (2005) and Swatevacharkul (2009). The *desire* (D) scale consists of nine items, which were all adapted from Hsu (2005). Metacognitive knowledge and metacognitive skills are subcomponents of metacognition. *Metacognitive knowledge* (MK) covers metacognitive knowledge about self (MKS), language (MKL), context (MKC), and the learning process (MKP). MKS consists of eight items from Cotterall (1995, 1999) and Hsu (2005). MKL is made up of seven items, which were adapted from Hsu (2005) and Dixon (2011). MKC contains six items, which were adapted from Hsu (2005), and MKP has six items from Cotterall (1999) and Hsu (2005). However, MKS, MKL, MKC, and MKP were not investigated separately, but together under the *metacognitive knowledge* scale. There are three *metacognitive skills* (MS – planning, monitoring, and evaluating). *Metacognitive skill – planning* (MSP) consists of seven items from Dang (2012) and Yang (2007). Items 18, 45, and 80 were added by us. *Metacognitive skill – monitoring* (MSM) has ten items which were adapted from Dang (2012) and Yang (2007). There are five items in metacognitive skill – evaluating (MSE), all of which were adapted from Dang (2012) and Yang (2007). These skills are examined on the metacognitive skills scale. The *freedom* (F) scale contains ten items which were all adapted from Chan et al. (2002).

### 4.3 Data collection and analytical procedures

After the survey was established, it was revised through discussions with our research team whose discussions, comments, and feedback were very helpful and contributed a great deal to the development of the survey. Back-translation method was used to enable the particpants to understand the questionnaire (see more in Sousa and Rojjanasrirat, 2011).

The questionnaire was translated into Vietnamese so that all the respondents, who are native speakers of Vietnamese, could understand all the contents of the survey and the validity of the data could be improved. The Vietnamese version was translated back into English with the help of a small group of peers. As part of this process, known as back-translation, it was sent to one Vietnamese-American in the US, to one PhD and two PhD candidates in Australia and New Zealand, and to three instructors who hold Master's degrees currently in Vietnam. All of them have expertise in ELT and have been working as instructors of English for many years. All the differences between the new English versions were critically reviewed, compared, and contrasted with the original English version. Clarification was requested on any ambiguous points and as a result, several minor word choice modifications were made. Finally, a trial version of the survey in Vietnamese was produced. It was emailed to several other ELT experts to read and provide remarks on face and content validity. They offered comments on the wording of the items in terms of meaningfulness and interpretability. We also sent the trial questionnaire to four Vietnamese undergraduates majoring in different fields, who did not participate in the study. It took them around 30 min to read and complete it. The trial showed that they did not encounter any difficulties in understanding the survey and that the design was friendly to its users. Therefore, no changes were made to that Vietnamese version and it was officially used in this study.

After obtaining ethical approval from the Institutional Review Board and permission from the universities, one of us, the first author, came to the classes to talk to the students about the study in terms of aims, significance, methods, and, importantly, ethical issues. The participants were fully informed that their responses would not be detrimental to them in any way and would be treated with confidentiality and utilized merely for research purposes. The printed questionnaires were delivered to the participants, and any questions regarding the research were satisfactorily elucidated. The participants spent approximately 20 min reading and completing the questionnaire. Of the 1,600 questionnaires that were distributed, 1,565 students completed them and returned them to us. 35 were discarded due to incompleteness and/or the students’ wishes. This represents a nearly 98% response rate.

The data collected was coded into SPSS Version 24.0, SPSS AMOS, and SmartPLS 3. SPSS AMOS and SmartPLS 3 used the SPSS input data. This study pondered five out of six aspects of validity, which were content, substantive, structural, external, and consequential. We delved into the reliablity based on Cronbach's alpha, composite reliability, average inter-item correlations, and rho_A.

## 5 Results

### 5.1 Validity

There is no statistical data representing content validity, but it is indicated via the process of defining LA, reviewing the literature, and generating items on the basis of sound arguments from the literature. We designed the survey to investigate aspects of LA. All the items were systematically reviewed in the literature, selected from well-established previous studies, changed to be culturally appropriate in the current context, and critically commented on by a group of ELT experts. Therefore, the instrument relatively covered the items it purported to cover, and it demonstrated content validity.

We examined the substantive aspect of validity on the basis of the need for empirical data on response consistencies (Messick, 1995). This was done through a review of both the international and national literature. Then, the gaps convincingly showed that there was a need for more empirical data on questionnaire validation and on how LA is perceived among a certain group of participants.

We used EFA and CFA to obtain evidence of the structural aspect of validity. EFA was necessary in this study to inspect the participants' responses to the questionnaire because the items on the LAPQ were borrowed, rewritten, added, and adapted from different questionnaires so that they would be culturally suited to the Vietnamese context. SPSS version 24.0 was utlized to provide results of EFA. According to Bartlett's test of sphericity, which assesses the significance of all the correlations in the correlation matrix, it was significantly appropriate to conducting a factor analysis (n = 780; χ^2^ = 27,614.745; d.f. = 3,741; p < 0.001). In addition, the Kaiser–Meyer–Olkin measure of sampling adequacy showed that the strength of the relationships between variables was “marvelous” to proceed with the analysis (KMO = .933) (Kaiser and Rice, 1974, p. 112). EFA was conducted with the support of PA using principal axis factoring because factors were assumed to be correlated (Crawford et al., 2010). Based on the comparison of eigenvalues between the parallel random data (95th percentile and average eigenvalues) and the actual one, Table 3 revealed that the first five actual eigenvalues were greater than those in both average and 95th percentile columns; as a results, five factors were extracted.

**Table 3:** Eigenvalues generated from PA.

Factors	Actual eigenvalues	Average eigenvalues	95th percentile eigenvalues
1	16.509	1.556	1.586
2	4.617	1.524	1.545
3	3.166	1.498	1.519
4	1.731	1.477	1.496
5	1.502	1.459	1.478
6	1.367	1.441	1.460
7	1.213	1.425	1.446

**Figure 3:**
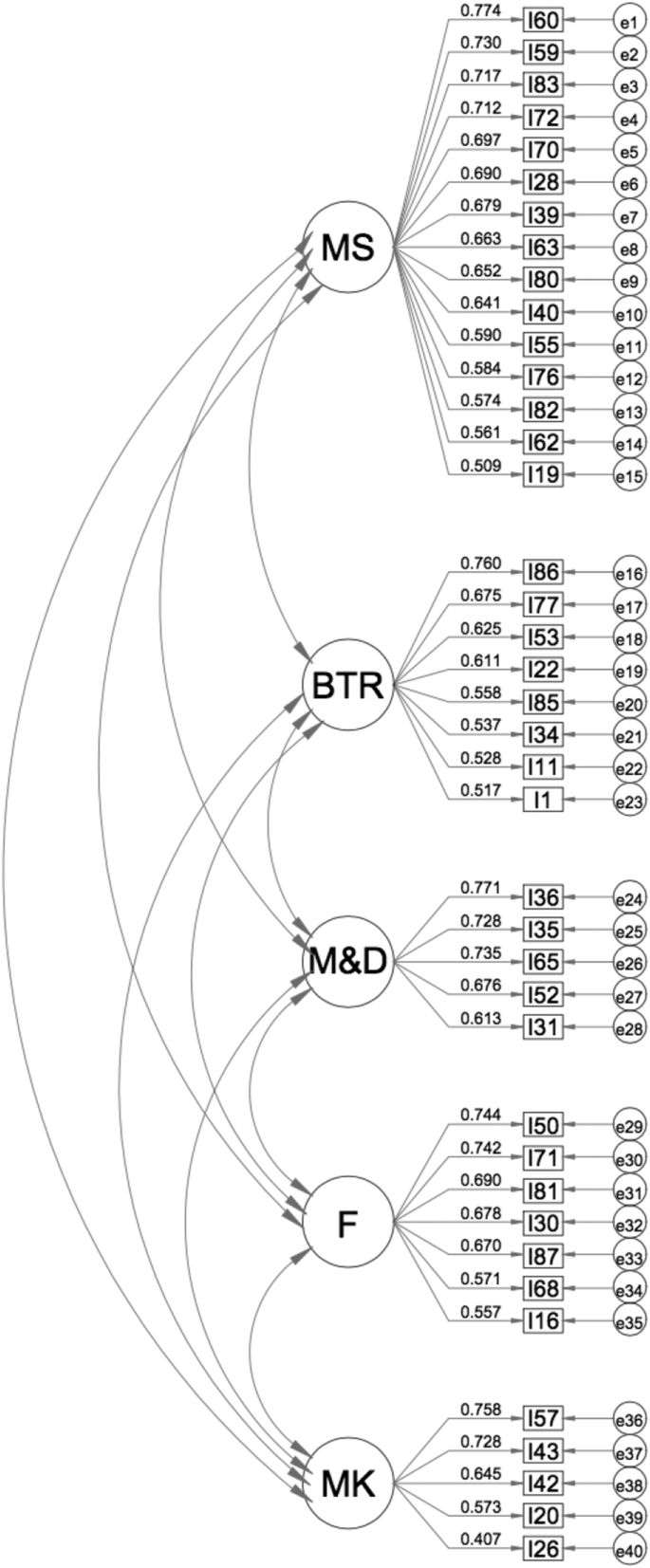
Model for the 40-item LAPQ

After iterative EFA using principal axis factoring and double oblimin due to the assumption of correlations among factors (Costello and Osborne, 2005), 47 items (i.e., items 2, 3, 4, 5, 6, 7, 8, 9, 10, 12, 13, 14, 15, 17, 18, 21, 23, 24, 25, 27, 29, 33, 34, 37, 38, 41, 44, 45, 46, 47, 48, 49, 51, 54, 56, 58, 61, 64, 66, 67, 69, 73, 74, 75, 78, 79, and 84) were excluded because of either ambiguous or low factor loadings. For instance, item 54 entitled “I learn English because I want to be as good at English as someone I know”, which iteratively had ambiguous loadings, was left out. This might be because taking someone else as an example to learn better was not preferred by students and it was not a type of their learning motivation. Some others initially intended to explore a scale (i.e., items 52, 55, 68, 76, and 85) were finally highly related to another scale and had high loadings. For example, although item 85 entitled “I need a lot of guidance in learning English” was intended for examining MK about self, its content was related to the notion of BTR and as a matter of fact, its loading was high in the factor of BTR. The final version consisted of 40 of the original 87 items and accounted for 51.637% of the variance (see Appendix C). The first factor referred to metacognitive skills (15 items), the second factor consisted of items tapping students' beliefs about teacher's role (eight items), the third factor delineated motivation and desire to learn English (five items), the fourth factor contained items referring to students' freedom (seven items), and the last factor elaborated on metacognitive knowledge (five items). CFA was performed on the revised 40-item questionnaire (n = 785), and maximum likelihood estimation was used to examine the model's parameters, absolute fit indices, and comparative fit indices. SPSS AMOS aided us in conducting CFA to examine the hypothesized model and calculate fit indices including RMSEA, TLI, CFI, and χ^2^/d.f. SmartPLS3 brought us with SRMR, RMS_theta, and NFI. Overall, the fit of the five-factor, 40-item model was not entirely satisfactory (χ^2^ = 1,633.966; d.f. = 367; χ^2^/d.f. = 4.45 < 5.0; p < 0.01; SRMR = 0.057 < 0.06; RMSEA = 0.047 < 0.05; RMS_theta = 0.104 < 0.12; NFI = 0.860 ≈ 0.9; TLI = 0.876 ≈ 0.9; CFI = 0.888 ≈ 0.9). Specifically, although Chi-square statistics, *p* value, SRMR, RMSEA, and RMS_theta suggest a reasonable fit to the students' responses, NFI, TLI, and CFI were slightly lower than the recommended value of 0.9. The standardized five-factor model is illustrated in Figure 3. Big circles represent latent variables, whereas the small ones show measurement errors associated with observed variables. Rectangles indicate the variables observed. Two-way arrows delineate correlations between two observed latent variables.

The external aspect of validity for the LAPQ was demonstrated by convergent and discriminant evidence. Convergent validity is confirmed by factor loadings, AVE, and CR. The statistics showed that all the items tested had acceptable factor loadings ranging from 0.407 to 0.774. AVE values of the scales ranged from 0.368 (BTR) to 0.502 (M&D). M&D, which achieved a high level of CR (0.834), had acceptable AVE values, which were more than 0.5, and the AVE for the other scales was below 0.5. However, the CR values for these scales were very high from 0.764 to 0.915. Therefore, it can be stated that convergent validity was established (see Table 4).

**Table 4:** Composite reliability and average variance extracted (AVE).

	**CR**	**AVE**
**BTR**	0.821	0.368
**F**	0.848	0.446
**M&D**	0.834	0.502
**MK**	0.764	0.403
**MS**	0.915	0.422

Abbreviations: BTR = beliefs about the teacher's role; F = freedom; M&D = motivation and desire; MK = metacognitive knowledge; MS = metacognitive skills.

Discriminant validity was indicated by three distinguishable criteria: the Fornell–Larcker criterion, cross-loadings, and the HTMT ratio. The data analysis on SPSS 24.0 and SmartPLS 3 showed that, as regards the first criterion, the square roots of the scales’ AVE were higher than those correlations of the scales with each other (see Table 5).

**Table 5:** Fornell–Larcker criterion (∗square root of AVE).

	**BTR**	**F**	**M&D**	**MK**	**MS**
**BTR**	0.607∗				
**F**	0.245	0.668∗			
**M&D**	0.299	0.382	0.708∗		
**MK**	0.295	0.298	0.338	0.635∗	
**MS**	0.223	0.583	0.470	0.400	0.650∗

With regard to the second criterion, all the scales had better correlations with themselves than with other scale variables (see Appendix D). In terms of the third criterion, HTMT ratios were all below 0.9 (see Table 6). Three points above mean that the discriminant validity was confirmed on all three different evaluations.

**Table 6:** Heterotrait-monotrait ratio of correlations (HTMT ratio).

	**BTR**	**F**	**M&D**	**MK**	**MS**
**BTR**					
**F**	0.288				
**M&D**	0.355	0.480			
**MK**	0.371	0.401	0.464		
**MS**	0.247	0.675	0.548	0.474	

The consequential aspect of validity emphasizes the score interpretation and potential consequences of LAPQ scores. We worked on the descriptive statistics and inferential statistics from the items to investigate how LA was perceived by the participants.

### 5.2 Reliability

The internal consistency (Cronbach's alpha) of the 40 items affirmed that the questionnaire had achieved excellent reliability, α = 0.902. The analyses of reliability are summarized in Table 7 below.

**Table 7:** Summary of reliability analysis.

	**Cronbach's α**	**rho_A**	**CR**
**BTR**	0.767	0.798	0.821
**F**	0.791	0.803	0.848
**M&D**	0.751	0.760	0.834
**MK**	0.633	0.677	0.764
**MS**	0.901	0.907	0.915

The reliability analysis demonstrated that almost all of the scales, BTR, MS, and M&D, achieved good and acceptable Cronbach's alpha coefficients and rho_A (α; rho_A > 0.7). The *metacognitive skills* field possessed the highest reliability level (α = 901, CR = 0.915, rho_A = 0.907). The MK scale had questionable alphas and rho_A (0.7 > α; rho_A ≥ 0.6). However, CR achieved a good value of 0.764, so the MK scale's reliability level was adequate. The average inter-item correlations among the final sub-scales ranged from 0.205 to 0.408 (Table 8), and that of the whole questionnaire was 0.354. These figures satisfied the criteria of good values (see more in Ferrell et al., 2000; Piedmont, 2014). Among 40 items, there were not any items suggested for deletion to increase reliability because bad items had been omitted during validity analyses above.

**Table 8:** Inter-item correlations for the sub-scales.

	**BTR**	**F**	**M&D**	**MK**	**MS**
**BTR**	1.000	0.279	0.206	0.205	0.295
**F**	0.279	1.000	0.363	0.340	0.373
**M&D**	0.206	0.363	1.000	0.391	0.408
**MK**	0.205	0.340	0.391	1.000	0.398
**MS**	0.295	0.373	0.408	0.398	1.000

## 6 Discussion

This study examined the reliability and validity aspects of the questionnaire, which aimed at investigating non-English major tertiary students’ perceptions of LA in language learning in a sample of Vietnamese students. The questionnaire development started with a critical review of the relevant literature, followed by a careful selection of items and an addition of appropriate items. The literature review enabled us to develop an operational definition of LA in this study. Despite the existence of a variety of definitions of LA, a specific definition would help researchers to clarify exactly what is developed and measured (Macaskill and Taylor, 2010). Autonomous learners in English learning, thus, hold rational beliefs about teacher roles, have both motivation and desire to learn the language, have sufficient metacognitive knowledge along with metacognitive skills, need freedom to take over their language learning and as a result, learn foreign languages effectively and fulfill their needs. Therefore, there were six aspects which made up six initial scales of the questionnaire. They specified beliefs about teacher role, motivation, metacognition (i.e., metacognitive knowledge and metacognitive skills), freedom, motivation, and desire.

In terms of validity, five aspects of validity from Messick (1995) framework were explored. They were content, substantive, structural, external, and consequential. Three of these including content, substantive, and consequential were satisfied and well-explained. The structural aspect of validity at the EFA level using PA was adequately fulfilled, and there was support for the five-factor, 40-item model that underlies the LAPQ. Six factors representing six scales at the beginning turned into five factors. Items under “motivation” and “desire” loaded into only one factor which was named “motivation and desire” in the end. This was in line with previous studies that investigated both motivation and desire as one category (Gardner, 1985; Hsu, 2005; Masgoret and Gardner, 2003). Indeed, the PA analysis of those five factors yielded some sufficient psychometric quality of the results. However, at the CFA level, the hypothesized model was not truly well-fitted to the data. The absolute fit indices were all good, but the comparative fit indices were close to the cut-off values. The external aspect was demonstrated through the confirmation of convergent and discriminant evidence. Those bodies of evidence were successfully obtained with the support of various values, such as CR, AVE, cross-loadings, and the Fornell–Larcker criterion.

After the validity analyses, the revised 40-item version of the questionnaire demonstrated an excellent level of overall Cronbach's alpha. All the scales had very good CR values as well as average inter-item correlations, good and acceptable values of internal consistency, and rho_A reliability. The evidence of discriminant and convergent validity as well as the average inter-item correlations contributed to strengthening the theoretical relationships which were positive among the components of LA in a model of structural and functional dynamics (Tassinari, 2012, 2015, 2018).

In conclusion, after the revisions, the new version of the questionnaire with 40 items achieved a solid level of reliability, and the majority of aspects of validity were tested in the current study, suggesting that it can be used to investigate students’ LA in tertiary language education.

## 7 Conclusion

The current study was devoted to establishing a survey of LA and notably to scrutinizing the reliability and validity of the LAPQ among non-English major undergraduates. On the whole, the LAPQ can be employed instantly by researchers and teachers or lecturers for two reasons. Firstly, the LAPQ showed sufficient evidence of reliability and validity, notwithstanding the fact that the hypothesized model of five factors was not properly confirmed. Hence, we urge future researchers to iterate as many validations as possible to see if the reliability and validity patterns, especially the model fit, are the same across various populations. Secondly, the LAPQ may yield useful results for some aspects of language learning processes for both educators and students so that they can reflect on themselves and make any improvements that may be necessary.

## 8 Research and theoretical implications

The present research has several implications for future studies. A specific operational definition offered was twofold. Firstly, we were able to develop the research instrument and validate it throughout the project. Secondly, the definition may serve as a useful reference for further studies on LA because it seemed to cover quite a few aspects of LA. Moreover, as an accurate method in factor analysis, PA which has not been widely used was employed in this study to accommodate retention of components and it is suggested that future studies can use PA more for analyzing factors. The study also contributes to the literature by validating a newly compiled questionnaire to explore LA among university students in a Vietnamese sample. The findings provide the first psychometric or validational evidence for the five-factor, 40-item LAPQ to examine students' perceptions of LA from different aspects in an Asian sample. Future research should build on the current findings to revalidate the scales for a more psychometrically sound questionnaire and to empirically explore the relationships among the sub-scales constituting the operational definition. Studies should be replicated to find out how it works in other contexts. More importantly, the study also provides researchers, educators, and teachers with a practical survey tool to attain a comprehensive overview of students' LA, which is seen to be a key to success at college and in life (Vansteenkiste et al., 2005). Then, they can help their students to raise their own awareness of LA in terms of which beliefs about teacher's role the students should adhere to, how teachers can enhance metacognitive proficiency, how they can give their students more freedom, and how students can develop the motivation and desire to learn languages. In this regard, those English learners will be empowered to learn foreign languages better and then use them better in the future.

## 9 Limitations

There exist some caveats in this study which call for further attention. The first is the proposed model of LA in this study, which did not show sufficient goodness-of-fit. This could be due to a failure to estimate the direct relationships between factors and items that led to model misspecification. Another possible explanation was that the scales should be validated in different contexts with various populations (see more in Teo, 2013). Students with different backgrounds may thus demonstrate differences in their LA perceptions. Moreover, it is widely accepted that responses depend on the target sample (Horwitz, 2008; Park, 2014). The same construct interpretations may vary among different groups, so there is no such thing as an absolutely validated questionnaire survey (Gu, 2018). It is, therefore, highly recommended that the questionnaire validation process be as iterative as possible. Secondly, although psychometric quality was amply ensured, one scale indicated moderate Cronbach's alpha and rho_A; thus, some revisions would be needed for future research. Thirdly, the researchers could not take on the generalizability aspect of validity in Messick (1995) framework of validity because this study recruited participants who are mostly from the northern and central regions of Vietnam. In addition, the universities are all in the north of the country, so those from other parts were not included in this research. The results can only be generalized through more research. Broadening the questionnaire to other populations can help determine the reliability of the tool for other groups (Habók & Magyar, 2018). It is also advisable to formulate and validate new versions of the LAPQ for high school students. This is because they need to be ready for LA at university as a transitional stage, which is a determinant of future academic and socio-economic status (Pham and White, 2018). Last but not least, to our best knowledge, there are various aspects of LA from previous studies, but those researchers only examined some aspects that are relevant to the context. Further studies will involve more explorations of other angles or corners of LA to enrich the literature.

## Declarations

### Author contribution statement

Son Van Nguyen: Conceived and designed the experiments; Performed the experiments; Analyzed and interpreted the data; Contributed reagents, materials, analysis tools or data; Wrote the paper.

Anita Habók: Contributed reagents, materials, analysis tools or data.

### Funding statement

This work was supported by the 10.13039/501100015763University of Szeged Open Access Fund (Grant number: 5035).

### Data availability statement

The data that has been used is confidential.

### Declaration of interests statement

The authors declare no conflict of interest.

### Additional information

No additional information is available for this paper.

## Footnotes

1 Retrieved from https://www.smartpls.com/documentation/algorithms-and-techniques/model-fit.
