# Supplementary material for: Designing and validating the learner autonomy perception questionnaire
Source: Heliyon. 2021 Apr 21;7(4):e06831. doi: 10.1016/j.heliyon.2021.e06831 (PMC8085706; doi:10.1016/j.heliyon.2021.e06831)
Supplement: Appendix [file mmc1.docx]

**Appendices**

**Appendix A**

**Learner Autonomy Perception Questionnaire**

The following statements are aimed at investigating your perception of learner autonomy. Please indicate the extent to which you agree or disagree with each of the statements about your English learning by ***circling the number that matches your opinion***. ***Number 0 is an example***.

1= Strongly disagree; 2= Disagree; 3= Neutral; 4=Agree; 5= Strongly agree

| **No.** | **STATEMENTS** | Strongly disagree | Disagree | Neutral | Agree | Strongly agree |
| --- | --- | --- | --- | --- | --- | --- |
| 0 | I like using Facebook to chat with my friends. | 1 | 2 | 3 | 4 | **➄** |
| 1 | The teachers should explain everything to us. | 1 | 2 | 3 | 4 | 5 |
| 2 | I learn English because it will help me to get a good job. | 1 | 2 | 3 | 4 | 5 |
| 3 | When it comes to any English tasks, I work very carefully to make sure I understand everything. | 1 | 2 | 3 | 4 | 5 |
| 4 | If I have any opportunities to use English outside class, I’ll use it most of the time and some Vietnamese, if necessary. | 1 | 2 | 3 | 4 | 5 |
| 5 | I have my own opinions about learning English and can defend them. | 1 | 2 | 3 | 4 | 5 |
| 6 | The most important part of learning a foreign language is learning grammar. | 1 | 2 | 3 | 4 | 5 |
| 7 | People in Vietnam who can speak English well have a better social status (e.g. they make more money, they are more educated, etc.). | 1 | 2 | 3 | 4 | 5 |
| 8 | I’m determined to achieve the target I’ve set for my English learning. | 1 | 2 | 3 | 4 | 5 |
| 9 | I change my learning content and target according to my needs. | 1 | 2 | 3 | 4 | 5 |
| 10 | I think about the methods I use to learn English and whether they are good. | 1 | 2 | 3 | 4 | 5 |
| 11 | The teachers should decide the objectives of my English courses. | 1 | 2 | 3 | 4 | 5 |
| 12 | I learn English because I want to pass exams. | 1 | 2 | 3 | 4 | 5 |
| 13 | I use my learning style effectively. | 1 | 2 | 3 | 4 | 5 |
| 14 | The teachers should evaluate my learning. | 1 | 2 | 3 | 4 | 5 |
| 15 | I try to listen to English regularly (songs, radio, TV, YouTube, Facebook, etc.). | 1 | 2 | 3 | 4 | 5 |
| 16 | I have chances to work with my classmates in activities in English class. | 1 | 2 | 3 | 4 | 5 |
| 17 | I check my English proficiency by taking English tests voluntarily. | 1 | 2 | 3 | 4 | 5 |
| 18 | Before doing any English tasks, I think about the knowledge I have of the topics involved. | 1 | 2 | 3 | 4 | 5 |
| 19 | I make good use of materials and resources when studying English. | 1 | 2 | 3 | 4 | 5 |
| 20 | To learn English well, it’s important to know one’s personality, motivation, personal needs, expectations, learning styles, strengths, weaknesses, etc., in English. | 1 | 2 | 3 | 4 | 5 |
| 21 | I try using other methods if one method of learning English doesn’t suit me. | 1 | 2 | 3 | 4 | 5 |
| 22 | The teachers should ensure my progress in learning English. | 1 | 2 | 3 | 4 | 5 |
| 23 | I learn English so that I can communicate with English speakers. | 1 | 2 | 3 | 4 | 5 |
| 24 | If my teacher wanted someone to do an extra English assignment, I’d definitely volunteer. | 1 | 2 | 3 | 4 | 5 |
| 25 | It’s important to understand every word when you read an English text. | 1 | 2 | 3 | 4 | 5 |
| 26 | There are a lot of opportunities to learn English in Vietnam. | 1 | 2 | 3 | 4 | 5 |
| 27 | I know how to set my own learning goals. | 1 | 2 | 3 | 4 | 5 |
| 28 | I carry out learning plans once they’ve been made. | 1 | 2 | 3 | 4 | 5 |
| 29 | I take advantage of opportunities to speak English. | 1 | 2 | 3 | 4 | 5 |
| 30 | I have chances to discuss learning issues with my classmates. | 1 | 2 | 3 | 4 | 5 |
| 31 | I’d like to have friends from English-speaking countries. | 1 | 2 | 3 | 4 | 5 |
| 32 | The teachers should decide how long to spend on each activity. | 1 | 2 | 3 | 4 | 5 |
| 33 | The teachers should ask us to share our views in class. | 1 | 2 | 3 | 4 | 5 |
| 34 | I learn English because it’s a required course at my university. | 1 | 2 | 3 | 4 | 5 |
| 35 | If English were not taught at my university, I’d try to take English classes somewhere else. | 1 | 2 | 3 | 4 | 5 |
| 36 | I’d like English to be used as much as possible in English class. | 1 | 2 | 3 | 4 | 5 |
| 37 | It’s necessary to know about English-speaking cultures to learn English well. | 1 | 2 | 3 | 4 | 5 |
| 38 | My classmates are active English learners. | 1 | 2 | 3 | 4 | 5 |
| 39 | I make my schedule so I’ll have enough time to study English. | 1 | 2 | 3 | 4 | 5 |
| 40 | I deal with things related to English but not necessarily related to English class. | 1 | 2 | 3 | 4 | 5 |
| 41 | The teachers should stimulate my interest in learning English. | 1 | 2 | 3 | 4 | 5 |
| 42 | I understand my own personality. | 1 | 2 | 3 | 4 | 5 |
| 43 | I’m responsible for the success of my English learning. | 1 | 2 | 3 | 4 | 5 |
| 44 | The university treats English as a very important subject. | 1 | 2 | 3 | 4 | 5 |
| 45 | Before doing homework or class work, I think about the skills I have to complete those types of tasks. | 1 | 2 | 3 | 4 | 5 |
| 46 | I actively participate in class activities. | 1 | 2 | 3 | 4 | 5 |
| 47 | I write in English (emails, a diary, my Facebook status, etc.). | 1 | 2 | 3 | 4 | 5 |
| 48 | I think about my progress in learning English. | 1 | 2 | 3 | 4 | 5 |
| 49 | I know how to find my own ways to practice English. | 1 | 2 | 3 | 4 | 5 |
| 50 | I can go see my teachers about my English learning. | 1 | 2 | 3 | 4 | 5 |
| 51 | I believe I have the ability to learn English successfully. | 1 | 2 | 3 | 4 | 5 |
| 52 | I learn English because it will help me to be successful in my studies. | 1 | 2 | 3 | 4 | 5 |
| 53 | The teachers should correct all my mistakes. | 1 | 2 | 3 | 4 | 5 |
| 54 | I learn English because I want to be as good at English as someone I know. | 1 | 2 | 3 | 4 | 5 |
| 55 | After I get my English work back, I always read it again to correct my mistakes. | 1 | 2 | 3 | 4 | 5 |
| 56 | I learn English because I want to please my family. | 1 | 2 | 3 | 4 | 5 |
| 57 | I know my strengths and weaknesses in learning English. | 1 | 2 | 3 | 4 | 5 |
| 58 | I feel my English teacher is like a friend. | 1 | 2 | 3 | 4 | 5 |
| 59 | I plan how I learn English. | 1 | 2 | 3 | 4 | 5 |
| 60 | I reflect on what I learn and look for something important. | 1 | 2 | 3 | 4 | 5 |
| 61 | I read English materials (books, notices, newspapers, online news, etc.). | 1 | 2 | 3 | 4 | 5 |
| 62 | I give myself a reward or treat when I do something well in English. | 1 | 2 | 3 | 4 | 5 |
| 63 | I notice my mistakes and use that information to improve. | 1 | 2 | 3 | 4 | 5 |
| 64 | The teachers should choose what activities to use to learn English in English class. | 1 | 2 | 3 | 4 | 5 |
| 65 | I learn English because I find it very interesting. | 1 | 2 | 3 | 4 | 5 |
| 66 | If there were an English club at my university, I’d be interested in joining. | 1 | 2 | 3 | 4 | 5 |
| 67 | It’s important to have excellent pronunciation in English. | 1 | 2 | 3 | 4 | 5 |
| 68 | We use a lot of English in English class at my university. | 1 | 2 | 3 | 4 | 5 |
| 69 | I know how to measure my progress. | 1 | 2 | 3 | 4 | 5 |
| 70 | I try to study English regularly even with limited time. | 1 | 2 | 3 | 4 | 5 |
| 71 | I have chances to ask the teachers questions when I don’t understand something. | 1 | 2 | 3 | 4 | 5 |
| 72 | I check to make sure I’ve understood what I need to learn. | 1 | 2 | 3 | 4 | 5 |
| 73 | I know how to check my work for mistakes. | 1 | 2 | 3 | 4 | 5 |
| 74 | The most important part of learning English is learning vocabulary. | 1 | 2 | 3 | 4 | 5 |
| 75 | Considering how I study English, I can honestly say that I do just enough to get by. | 1 | 2 | 3 | 4 | 5 |
| 76 | I put great effort into learning English. | 1 | 2 | 3 | 4 | 5 |
| 77 | The teachers should choose what materials to use to learn English in English class. | 1 | 2 | 3 | 4 | 5 |
| 78 | The most important part of learning English is translating from Vietnamese. | 1 | 2 | 3 | 4 | 5 |
| 79 | I know how to plan my English learning. | 1 | 2 | 3 | 4 | 5 |
| 80 | Before I do class work or homework, I analyze what’s required. | 1 | 2 | 3 | 4 | 5 |
| 81 | I have chances to make suggestions to the teachers. | 1 | 2 | 3 | 4 | 5 |
| 82 | I try to complete things I’ve decided to do. | 1 | 2 | 3 | 4 | 5 |
| 83 | I set my goals in learning English. | 1 | 2 | 3 | 4 | 5 |
| 84 | It’s important to understand every word when you listen to English. | 1 | 2 | 3 | 4 | 5 |
| 85 | I need a lot of guidance in learning English. | 1 | 2 | 3 | 4 | 5 |
| 86 | The teachers should set my learning goals. | 1 | 2 | 3 | 4 | 5 |
| 87 | I have chances to do English self-study with friends. | 1 | 2 | 3 | 4 | 5 |

**Appendix B**

**Items and their sources**

| **Number** | **Scale** | | **Statements** | **Sources** |
| --- | --- | --- | --- | --- |
| 1 | **Beliefs about teachers’ role** | | 1. The teachers should explain everything to us.  11. The teachers should decide the objectives of my English courses.  14. The teachers should evaluate my learning.  22. The teachers should ensure my progress in learning English.  32. The teachers should decide how long to spend on each activity.  33. The teachers should ask us to share our views in class.  41. The teachers should stimulate my interest in learning English.  53. The teachers should correct all my mistakes.  64. The teachers should choose what activities to use to learn English in English class.  77. The teachers should choose what materials to use to learn English in English class.  86. The teachers should set my learning goals. | Adapted from Chan et al. (2002), Le (2013), and Ming and Alias (2007) |
| 2 | **Motivation and desire** | **Motivation** | 2. I learn English because it will help me to get a good job.  12. I learn English because I want to pass exams.  23. I learn English so that I can communicate with English speakers.  34. I learn English because it’s a required course at my university.  52. I learn English because it will help me to be successful in my studies.  54. I learn English because I want to be as good at English as someone I know.  56. I learn English because I want to please my family.  65. I learn English because I find it very interesting. | Adapted from Hsu (2005) and Swatevacharkul (2009)  No. 65: Authors’ addition |
|  |  | **Desire** | 3. When it comes to any English tasks, I work very carefully to make sure I understand everything.  4. If I have any opportunities to use English outside class, I’ll use it most of the time and some Vietnamese, if necessary.  24. If my teacher wanted someone to do an extra English assignment, I’d definitely volunteer.  31. I’d like to have friends from English-speaking countries.  35. If English were not taught at my university, I’d try to take English classes somewhere else.  36. I’d like English to be used as much as possible in English class.  55. After I get my English work back, I always read it again to correct my mistakes.  66. If there were an English club at my university, I’d be interested in joining.  75. Considering how I study English, I can honestly say that I do just enough to get by. | Adapted from Hsu (2005) |
| 3 | **Metacognitive knowledge in ELT** | **About self as a learner** | 5. I have my own opinions about learning English and can defend them.  13. I use my learning style effectively.  42. I understand my own personality.  43. I’m responsible for the success of my English learning.  51. I believe I have the ability to learn English successfully.  57. I know my strengths and weaknesses in learning English.  76. I put great effort into learning English.  85. I need a lot of guidance in learning English. | Adapted from Cotterall (1995, 1999) and Hsu (2005) |
|  |  | **About subject matter** | 6. The most important part of learning a foreign language is learning grammar.  25. It’s important to understand every word when you read an English text.  37. It’s necessary to know about English-speaking cultures to learn English well.  67. It’s important to have excellent pronunciation in English.  74. The most important part of learning English is learning vocabulary.  78. The most important part of learning English is translating from Vietnamese.  84. It’s important to understand every word when you listen to English. | Adapted from Dixon (2011) and Hsu (2005) |
|  |  | **About the learning context** | 7. People in Vietnam who can speak English well have a better social status (e.g. they make more money, they are more educated, etc.).  26. There are a lot of opportunities to learn English in Vietnam.  38. My classmates are active English learners.  44. The university treats English as a very important subject.  58. I feel my English teacher is like a friend.  68. We use a lot of English in English class at my university. | Adapted from Hsu (2005) |
|  |  | **About the learning process** | 20. To learn English well, it’s important to know one’s personality, motivation, personal needs, expectations, learning styles, my strengths, weaknesses, etc., in English.  27. I know how to set my own learning goals.  49. I know how to find my own ways to practice English.  69. I know how to measure my progress.  73. I know how to check my work for mistakes.  79. I know how to plan my English learning. | Adapted from  Cotterall (1999) and Hsu (2005) |
| 4 | **Metacognitive skills in ELT** | **Planning** | 8. I’m determined to achieve the target I’ve set for my English learning.  18. Before doing any English tasks, I think about the knowledge I have of the topics involved.  39. I make my schedule so I’ll have enough time to study English.  45. Before doing any English tasks, I think about the skills I have to complete those types of tasks.  59. I plan how I learn English.  80. Before I do class work or homework, I analyze what’s required.  83. I set my goals in learning English. | Adapted from Dang (2012) and Yang (2007);  Nos. 18, 45, 80: Authors’ addition |
|  |  | **Monitoring** | 9. I change my learning content and target according to my needs.  19. I make good use of materials and resources when studying English.  21. I try using other methods if one method of learning English doesn’t suit me.  28. I carry out learning plans once they’ve been made.  40. I deal with things related to English but not necessarily related to English class.  46. I actively participate in class activities.  63. I notice my mistakes and use that information to improve.  70. I try to study English regularly even with limited time.  72. I check to make sure I’ve understood what I need to learn.  82. I try to complete things I’ve decided to do. | Adapted from Dang (2012) and Yang (2007) |
|  |  | **Evaluating** | 10. I think about the methods I use to learn English and whether they are good.  17. I check my English proficiency by taking English tests voluntarily.  48. I think about my progress in learning English.  60. I reflect on what I learn and look for something important.  62. I give myself a reward or treat when I do something well in English. | Adapted from Dang (2012) and Yang (2007) |
| 5 | **Freedom** | | 15. I try to listen to English regularly (songs, radio, TV, YouTube, Facebook, etc.).  16. I have chances to work with my classmates in activities in English class.  29. I take advantage of opportunities to speak English.  30. I have chances to discuss learning issues with my classmates.  47. I write in English (emails, a diary, my Facebook status, etc.).  50. I can go see my teachers about my English learning.  61. I read English materials (books, notices, newspapers, online news, etc.).  71. I have chances to ask the teachers questions when I don’t understand something.  81. I have chances to make suggestions to the teachers.  87. I have chances to do English self-study with friends. | Adapted from Chan et al. (2002) |

**Appendix C**

**Factor solution, and corresponding LAPQ items**

| **Factors and items** | **Factor loadings** | | | | |
| --- | --- | --- | --- | --- | --- |
|  | **1** | **2** | **3** | **4** | **5** |
| **Metacognitive skills**  I60. I reflect on what I learn and look for something important. | .774 |  |  |  |  |
| I59. I plan how I learn English. | .730 |  |  |  |  |
| I83. I set my goals in learning English. | .717 |  |  |  |  |
| I72. I check to make sure I’ve understood what I need to learn. | .712 |  |  |  |  |
| I70. I try to study English regularly even with limited time. | .697 |  |  |  |  |
| I28. I carry out learning plans once they’ve been made. | .690 |  |  |  |  |
| I39. I make my schedule so I’ll have enough time to study English. | .679 |  |  |  |  |
| I63. I notice my mistakes and use that information to improve. | .663 |  |  |  |  |
| I80. Before I do class work or homework, I analyze what’s required. | .652 |  |  |  |  |
| I40. I deal with things related to English but not necessarily related to English class. | .641 |  |  |  |  |
| I55. After I get my English work back, I always read it again to correct my mistakes. | .590 |  |  |  |  |
| I76. I put great effort into learning English. | .584 |  |  |  |  |
| I82. I try to complete things I’ve decided to do. | .574 |  |  |  |  |
| I62. I give myself a reward or treat when I do something well in English. | .561 |  |  |  |  |
| I19. I make good use of materials and resources when studying English. | .509 |  |  |  |  |
| **Beliefs about teacher’s role**  I86. The teachers should set my learning goals. |  | .760 |  |  |  |
| I77. The teachers should choose what materials to use to learn English in English class. |  | .675 |  |  |  |
| I53. The teachers should correct all my mistakes. |  | .625 |  |  |  |
| I22. The teachers should ensure my progress in learning English. |  | .611 |  |  |  |
| I85. I need a lot of guidance in learning English. |  | .558 |  |  |  |
| I32. The teachers should decide how long to spend on each activity. |  | .537 |  |  |  |
| I11. The teachers should decide the objectives of my English courses. |  | .528 |  |  |  |
| I1. The teachers should explain everything to us. |  | .517 |  |  |  |
| **Motivation and desire**  I36. I’d like English to be used as much as possible in English class. |  |  | .771 |  |  |
| I35. If English were not taught at my university, I’d try to take English classes somewhere else. |  |  | .738 |  |  |
| I65. I learn English because I find it very interesting. |  |  | .735 |  |  |
| I52. I learn English because it will help me to be successful in my studies. |  |  | .676 |  |  |
| I31. I’d like to have friends from English-speaking countries. |  |  | .613 |  |  |
| **Freedom**  I50. I can go see my teachers about my English learning. |  |  |  | .744 |  |
| I71. I have chances to ask the teachers questions when I don’t understand something. |  |  |  | .742 |  |
| I81. I have chances to make suggestions to the teachers. |  |  |  | .690 |  |
| I30. I have chances to discuss learning issues with my classmates. |  |  |  | .678 |  |
| I87. I have chances to do English self-study with friends. |  |  |  | .670 |  |
| I68. We use a lot of English in English class at my university. |  |  |  | .571 |  |
| I16. I have chances to work with my classmates in activities in English class. |  |  |  | .557 |  |
| **Metacognitive knowledge**  I57. I know my strengths and weaknesses in learning English. |  |  |  |  | .758 |
| I43. I’m responsible for the success of my English learning. |  |  |  |  | .728 |
| I42. I understand my own personality. |  |  |  |  | .645 |
| I20. To learn English well, it’s important to know one’s personality, motivation, personal needs, expectations, learning styles, my strengths, weaknesses, etc., in English. |  |  |  |  | .573 |
| I26. There are a lot of opportunities to learn English in Vietnam. |  |  |  |  | .407 |

**Appendix D**

**Cross-loadings of the items**

|  | BTR | F | M & D | MK | MS |
| --- | --- | --- | --- | --- | --- |
| I1 | 0.517 | 0.095 | 0.036 | 0.087 | 0.045 |
| I11 | 0.528 | 0.068 | 0.081 | -0.002 | 0.056 |
| I16 | 0.069 | 0.557 | 0.303 | 0.141 | 0.264 |
| I19 | 0.107 | 0.297 | 0.261 | 0.132 | 0.509 |
| I20 | 0.291 | 0.123 | 0.193 | 0.573 | 0.156 |
| I22 | 0.611 | 0.045 | 0.131 | 0.193 | 0.121 |
| I26 | 0.006 | 0.156 | 0.126 | 0.407 | 0.139 |
| I28 | 0.075 | 0.401 | 0.189 | 0.186 | 0.690 |
| I30 | 0.185 | 0.678 | 0.350 | 0.265 | 0.412 |
| I31 | 0.170 | 0.225 | 0.613 | 0.267 | 0.248 |
| I32 | 0.537 | 0.103 | 0.171 | 0.111 | 0.005 |
| I35 | 0.144 | 0.198 | 0.735 | 0.245 | 0.314 |
| I36 | 0.285 | 0.264 | 0.771 | 0.211 | 0.391 |
| I39 | 0.223 | 0.407 | 0.372 | 0.423 | 0.679 |
| I40 | 0.056 | 0.294 | 0.412 | 0.207 | 0.641 |
| I42 | 0.108 | 0.164 | 0.071 | 0.645 | 0.187 |
| I43 | 0.265 | 0.239 | 0.324 | 0.728 | 0.284 |
| I50 | 0.171 | 0.744 | 0.313 | 0.234 | 0.488 |
| I52 | 0.253 | 0.267 | 0.676 | 0.313 | 0.218 |
| I53 | 0.625 | 0.064 | 0.159 | 0.169 | 0.130 |
| I55 | 0.308 | 0.391 | 0.347 | 0.156 | 0.590 |
| I57 | 0.182 | 0.235 | 0.258 | 0.758 | 0.399 |
| I59 | 0.138 | 0.387 | 0.265 | 0.320 | 0.647 |
| I60 | 0.139 | 0.467 | 0.337 | 0.377 | 0.774 |
| I62 | 0.045 | 0.354 | 0.107 | 0.161 | 0.561 |
| I63 | 0.138 | 0.385 | 0.287 | 0.254 | 0.663 |
| I65 | 0.194 | 0.370 | 0.735 | 0.184 | 0.454 |
| I68 | 0.212 | 0.571 | 0.275 | 0.239 | 0.268 |
| I70 | 0.088 | 0.365 | 0.364 | 0.210 | 0.697 |
| I71 | 0.194 | 0.742 | 0.199 | 0.177 | 0.467 |
| I72 | 0.152 | 0.490 | 0.404 | 0.284 | 0.712 |
| I76 | 0.108 | 0.319 | 0.239 | 0.227 | 0.584 |
| I77 | 0.675 | 0.239 | 0.266 | 0.297 | 0.213 |
| I80 | 0.188 | 0.365 | 0.271 | 0.277 | 0.652 |
| I81 | 0.194 | 0.690 | 0.156 | 0.143 | 0.392 |
| I82 | 0.168 | 0.347 | 0.251 | 0.211 | 0.574 |
| I83 | 0.191 | 0.351 | 0.374 | 0.345 | 0.717 |
| I85 | 0.558 | 0.146 | 0.149 | 0.147 | 0.117 |
| I86 | 0.760 | 0.244 | 0.257 | 0.201 | 0.203 |
| I87 | 0.103 | 0.670 | 0.165 | 0.167 | 0.377 |
